# Skin protection creams in medical settings: successful or evil?

**DOI:** 10.1186/1745-6673-3-15

**Published:** 2008-07-25

**Authors:** Emmanuelle Xhauflaire-Uhoda, Elena Macarenko, Raphaël Denooz, Corinne Charlier, Gérald E Piérard

**Affiliations:** 1 Laboratory of Skin Bioengineering and Imaging, Department of Dermatopathology, Liège, Belgium; 2 Department of Clinical Toxicology, University Hospital of Liège, Liège, Belgium

## Abstract

**Background:**

Chronic exposure to mild irritants including cleansing and antiseptic products used for hand hygiene generates insults to the skin. To avoid unpleasant reactions, skin protection creams are commonly employed, but some fail to afford protection against a variety of xenobiotics. In this study, two skin protection creams were assayed comparatively looking for a protective effect if any against a liquid soap and an alcohol-based gel designed for hand hygiene in medical settings.

**Methods:**

Corneosurfametry and corneoxenometry are two in vitro bioessays which were selected for their good reproducibility, sensitivity and ease of use. A Kruskal-Wallis ANOVA test followed by the Dunn test was realized to compare series of data obtained.

**Results:**

Significant differences in efficacy were obtained between the two assayed skin protection creams. One of the two tested creams showed a real protective effect against mild irritants, but the other tested cream presented an irritant potential in its application with mild irritants.

**Conclusion:**

The differences observed for the two tested skin protection creams were probably due to their galenic composition and their possible interactions with the offending products. As a result, the present in vitro bioassays showed contrasted effects of the creams corresponding to either a protective or an irritant effect on human stratum corneum.

## Introduction

The regular and repetitive use of cleansing and antiseptic products for hand hygiene in medical settings is typically at risk for inducing irritant contact dermatitis (ICD) [1-3]. Indeed, repeat chemical aggressions generate subclinical alterations of the stratum corneum (SC), and impair the natural barrier against various chemical xenobiotics and microorganisms. As a result, skin becomes imperceptibly harsh and ICD ensues.

In order to minimize the risk for developing ICD, prophylactic measures are offered including the application of skin protection creams (SPC) [4]. SPC are marketed for preventing or reducing the adsorption, penetration and absorption of irritants into the skin [4-6]. In practice, their use remains subject to a lively debate. In general, there is a lack of evidence of their efficacy. Some authors even suggest that inappropriate SPC applications are prone to induce additional irritation rather than benefit [7-9].

Various in vivo and vitro methods were developed in recent times for evaluating SPC efficacy [10-16]. Each of the methods probably presents some advantages but also disadvantages. In vitro methods are generally recommended for screening SPC efficacy because of their ease, speed, and safety. Indeed, the interpretation of in vivo testing may be clouded by large inter-individual differences.

Two bioassays, namely corneosurfametry and corneoxenometry [17-24], were designed for comparing SPC efficacy. Both methods entail collection of human SC to serve as substrate for testing the ex-vivo reactivity of xenobiotics with human tissue. Any noxious xenobiotic is placed at a given concentration in contact with the SC for a defined period of time. After rinsing, specific dyes are applied to the samples. The staining intensity is proportional to the removal and/or degradation of SC proteins and lipids. The colour of the samples is measured by reflectance colorimetry. The recorded value is indicative of the severity of the damage induced by the xenobiotic to the SC or, conversely, can be representative of the effect brought by a SPC [21].

The aim of the present study was to compare the efficacy of two SPC using corneosurfametry and corneoxenometry. The potentially noxious products were regular cleansing and antiseptic products used for hand hygiene in medical settings.

## Materials and methods

This study was performed in accordance with the Declaration of Helsinki and its subsequent amendments. Informed consent was obtained after the nature of the procedure had been fully explained. A total of 18 adults of both genders aged 18 to 55 years were enrolled. In each subject a series of 7 cyanoacrylate skin surface strippings (CSSS) were harvested from the volar aspect of each forearm. Their size reached 1.5* 3 cm.

The range of products tested in this study was prepared by Naqi (Halen, Belgium). There were designed to be used for hand hygiene. The specific claims for these products were rapid cleansing, disinfection and repair of the skin barrier. They presented as an alcohol-based gel and a liquid soap. Two SPC identified as SPC 1 and SPC 2 were assayed. The specific product compositions are listed in Table 1.

**Table 1:** Composition of the Naqi products used in the study.

**Alcohol gel**
Alcool, Aqua, Polyquaternium-37, Glycerin, Panthenol, PEG-7 glyceryl cocoate, Cyclomethicone, PPG-15-stearyl ether, Parfum, Lactic acid
**Liquid soap**
Aqua, Sodium laureth sulphate, Sodium laureth-11 carboxylate, PEG-7 glyceryl cocoate, PEG-4-rapeseedamide, Sodium chloride, Polysorbate 21, Lauryldimonium Hydroxypropyl, Hydrolyzed Wheat Protein, Laureth-10, Panthenol, Laureth-4, PEG-150 Distearate, Allantoin, Parfum, Phenoxyethanol, Methylparaben, Ethylparaben, Propylparaben, Butylparaben, Disodium EDTA
**Skin protection cream 1**
Aqua, Butyrospermum Parkii, Cetearyl Octanoate, Propylene Glycol, Sorbitan stearate, Dimethicone, Cetearylalcohol, Cyclomethicone, Trimethylsiloxysilicate, PPG-(15)-stearyl ether, Panthenol, Bisabolol, Allantoin, Sucrose cocoate, Xhantan Gum, Methylparaben, Propilparaben, Imidiazolidinyl Urea, Propylapraben, Imidazolidinyl Urea, Parfum, Tocopherol, EDTA
**Skin protection cream 2**
Aqua, Butyrospermum Parkii, Simmondsia Chinensis, Oxidized Corn Oil, Pentaerythrityl Tetracaprylate/Caprate, Glycerin, Cetyl esters, Octyl Stearate, Cetearylalcohol, Sucrose laurate, Sucrose Erucate, Bisabolol, Phenoxyethanol, Ethylhexylglycerin, Carbomer, Sodium Hydroxide, Trisodium Ethylenediamine Disuccinate, Inulin Lauryl Carbamate, Xanthan Gum, Parfum, Tocopherol.

Five CSSS harvested from five different volunteers were used for testing each potentially irritant xenobiotic. In order to mimick the regular use of these products, the liquid soap was used at a 1:1 water dilution, and the alcohol-based gel was used as a neat formulation. Each of the 5 CSSS was dipped for 10 min into a flask containing one of the given xenobiotics. After rinsing in running tap water, they were air-dried and stained for 1 min in a 30% hydro-alcoholic solution of toluidine blue and basic fuschin. The colour of the CSSS was measured by reflectance colorimetry in the CIELab system using a Chroma Meter^® ^CR 400 (Minolta, Osaka, Japan). The colorimetric index of mildness (CIM), representing the staining intensity, was calculated as previously described [23,24] following CIM = L* – Chroma C*

Previous studies indicated that CIM value decreased with increasing chemical alteration of the corneocytes. CIM reached about 65 – 70 for water-treated CSSS, and decreased down to zero or below with increasing aggressiveness of products against corneocytes [17].

In addition, the alcohol-based gel and the liquid soap were tested singly following repetitive dipping contacts. A 10-min contact time was secured and repeated 4 times. A 10-min rest period was respected between successive dipping procedures. During each rest period products were rinsed under running tap water for 1 min followed by air drying at room temperature. A similar design was followed combining successive applications of the liquid soap followed by the alcohol-based gel in a 4 repeat contact procedure.

In order to assess BC efficacy and their skin tolerance, a beforehand uniform SPC application was performed on the CSSS before initiating the ex-vivo cumulative irritancy tests described here above. SPC 1 and SPC 2 were tested on different series of CSSS for evaluating their protective effect against repetitive use the liquid soap and the alcohol-based gel. Controls consisted in similar testing without BC applications.

### Statistical analysis

All statistical evaluations were performed using the Instat 2.01, 1993 GraphPad software Macintosh. The Shapiro-Wilk test was used to assess the possible normality of data distribution. Due to the non Gaussian distribution of CIM values, they were summarized as medians and ranges. Comparisons between series of data were made using the unpaired non-parametric Kruskal-Wallis ANOVA test followed by the Dunn test. A p-value lower than 0.05 was considered statistically significant.

## Results

### Ex vivo cumulative irritancy test

All CIM values obtained after a single or repeat contacts with the liquid soap were above 40 indicating a good skin tolerance (Table 2). There was no significant difference with increasing the number of contacts with the liquid soap. Only a trend in median CIM decrease was yielded after 3 and 4 contacts. CIM median values in the range 30–45 yielded for the alcohol-based gel suggested a fairly good skin tolerance (Table 2). No statistical difference was yielded with increasing the number of applications. CIM values for the alcohol-based gel were lower than those obtained for the liquid soap. Indeed, some statistical differences were yielded between CIM values of the liquid soap and the alcohol-based gel (Table 3a).

**Table 2:** CIM data (median and range) obtained following a single or repetitive 10-min contact time with the liquid soap, the alcohol-based gel and both products successively

**Number of** ** contacts**	**Liquid soap**		**Alcohol-based gel**		**Liquid soap and alcohol-** **based gel**	
	**Median**	**Range**	**Median**	**Range**	**Median**	**Range**

1	61.7	42.3 – 71.3	32.9	8.9 – 39.9	21.4	-2.9 – 27.1
2	61.8	51.2 – 69.1	37	8.9 – 48.5	22.3	6.5 – 44.1
3	56.3	44.5 – 80.9	45.6	-5.9 – 53.2	26.9	10.2 – 33.5
4	58.4	43.9 – 66.8	40.1	24.9 – 67.1	30.7	-13.7 – 59.7

**Table 3:** 

a. Statistical comparison between CIM data after a single or several contacts with the alcohol-based gel and the liquid soap.				
**Liquid soap** **number of** ** contacts**	**Alcool-based gel** ** Number of contacts**			

	**1**	**2**	**3**	**4**

1	***	***	*	*
2	***	***	**	**
3	***	***	NS	*
4	***	**	NS	NS

b. Statistical comparison between CIM data after a single or several contacts with the combination of liquid soap with alcohol gel (LS+AG) and the liquid soap.				

**Liquid soap** **number of ** ** contacts**	**Liquid soap and alcohol-based gel** ** Number of contacts**			

	**1**	**2**	**3**	**4**
1	***	***	**	NS
2	***	***	***	*
3	***	**	**	NS
4	***	**	**	NS

* p < 0.05 ** p < 0.01 *** p < 0.001 NS: not significant.

* p < 0.05 ** p < 0.01 *** p < 0.001 NS: not significant.

Successive combined applications of the liquid soap and the alcohol-based gel resulted in a fair skin tolerance with CIM median values ranging 20–30 (Table 2). Surprisingly, the CIM median values raised with increasing the number of combined applications (Table 2). However, the range in CIM values got wider revealing large interindividual variability. A significant difference (p < 0.01) was yielded between one and four contacts with the combination of products. CIM values obtained after successive contacts with the liquid soap and the alcohol-based gel were significantly lower than the CIM values obtained following contact with the liquid soap alone (Table 3b).

### Skin protection cream 1 efficacy

SPC 1 was applied onto CSSS before repeat applications of the liquid soap. (Table 4). Compared to the absence of SPC 1 application (Table 2), the median CIM values surprisingly dropped after SPC 1 application showing, however, no significant change. On the overall, CIM median values remained in the range 35–50 suggesting a fairly good skin tolerance when SPC 1 was applied before the liquid soap. However, the range of CIM values became broader, indicating that the CSSS harvested from some volunteers were reactive to this combination of products.

**Table 4:** CIM data (median and range) obtained following a single or repetitive 10-min contact time with the liquid soap and/or the alcohol-based gel following beforehand one single homogeneous application of SPC1 on the CSSS.

**Number of** ** contacts**	**SPC 1 + liquid soap**		**SPC 1 + alcohol-based** ** gel**		**SPC 1 + liquid soap** ** and alcohol-based gel**	
	**Median**	**Range**	**Median**	**Range**	**Median**	**Range**

1	37.4	3.7 – 63.3	13.15	-15.5 – 60.8	21.34	0.2 – 32.3
2	47.5	2.9 – 83.5	15.4	-9.9 – 53.9	10.7	-22.7 – 31.6
3	49.7	14.3 – 64.3	18	-6.3 – 67.2	-3.1	-23 – 32.9
4	42.9	15.9 – 68.4	15.5	-17.5 – 55.1	1.5	-33.4 – 32.2

In another assessment, SPC 1 was applied before repeat applications of the alcohol-based gel (Table 4). A marked decrease in CIM median values was observed. Indeed, they dropped below 20 indicating some damage to the SC. A significant difference (p < 0.05) was reached at the fourth application of the alcohol-based gel when SPC1 has been applied or not beforehand (Tables 2 and 4).

The same procedure was performed with SPC 1 application before repeat applications of both the liquid soap and the alcohol-based gel (Table 4). A decrease in CIM median values was observed corresponding to an irritant potential of this combination. Significant differences between CIM values were yielded at the third application (p < 0.05) and at the fourth application (p < 0.01) when comparing SPC 1 application or not (Tables 2 and 4).

### Skin protection cream 2 efficacy

SPC 2 was applied onto CSSS before repeat applications of the liquid soap. (Table 5). The CIM median values increased when SPC 2 was applied beforehand. A significant difference (p < 0.01) was reached for one single application of the liquid soap (Tables 2 and 5). When repeat applications of the liquid soap were performed, no significant difference was yielded. Interestingly enough the skin tolerance of this combination of products appeared good with a very high CIM median value above 70.

**Table 5:** CIM data (median and range) obtained following a single or repetitive 10-min contact time with the liquid soap and/or the alcohol-based gel following beforehand one homogeneous application of SPC2 on the CSSS.

**Number of ** **contacts**	**SPC 2 + liquid soap**		**SPC 2 + alcohol-based** ** gel**		**SPC2 + Liquid soap and alcohol-based gel** ** alcohol-based gel**	
	**Median**	**Range**	**Median**	**Range**	**Median**	**Range**

1	77	64.4 – 84	56.42	33 – 85	45.9	38.6 – 59.5
2	73.1	36 – 82	42.7	-2.8 – 76	34.7	27.3 – 40.3
3	74.2	6.9 – 81.2	48.9	18.3 – 68.7	32.6	20.2 – 43.8
4	71.3	14.5 – 79.4	41.3	2.2 – 70.4	34.8	13 – 44.2

SPC 2 was also applied on CSSS before repeat applications of the alcohol-based gel (Table 5). The CIM median values were above 40, suggesting an overall good skin tolerance. SPC 2 application showed a significant improvement (p < 0.01) in the skin tolerance for a single application of the alcohol-based gel.

SPC 2 was applied before repeat applications of the combination of the liquid soap and the alcohol-based gel (Table 5). Compared to controls (Table 2), the CIM values were significantly improved when SPC 2 was applied onto CSSS (p < 0.001) for one application and for two applications of the offending products (p < 0.01). Starting from the third application, only a protective trend of SPC 2 was highlighted.

## Discussion

In the present study, cumulative irritancy tests were performed on CSSS for testing SPC efficacy. The bioassays were conducted in a way close to realistic conditions encountered at the workplace in medical settings. The rating methods were performed against controls in absence of SPC. The validity of the bioassays was previously established [17-24]. In general, the SC response in corneosurfametry and corneoxenometry shows some interindividual variability related in part to the perception of sensitive skin [25]. Similar variations in data range have been reported for other methods testing SPC [11]. These findings highlight the difficulty in rating the clinical value of SPC because the interindividual variability shown by different models share some relevance to the in vivo situation.

There are two main conceptual ways for ensuring that irritants and allergens do not penetrate into the skin. Some SPC are expected to prevent the penetration of the xenobiotic by a shielding mechanism. Otherwise, the offending compounds can be sequestrated and neutralized by the SPC in order to avoid their uptake by the SC. This latter mechanism can involve some chemical interactions between the xenobiotic and some SPC compounds.

The liquid soap and the alcohol-based gel were formulated in order to be mild for the skin. Their application on CSSS indicated a good skin tolerance. The liquid soap was indeed composed of non-ionic surfactants, selected for their good compatibility with the skin [1]. Moreover, allantoin, panthenol and hydrolysed wheat protein (Table 1) claim anti-irritant properties [26,27] that could further increase the global skin tolerance. The alcohol-based gel was similarly well tolerated on CSSS. This was probably related to the presence of panthenol and other moisturizing compounds like glycerol and lactic acid [27-29]. On the overall, a lower skin tolerance was observed for the alcohol-based gel compared to the liquid soap in their repeat applications. This finding is at variance with other works [3,30] suggesting that alcohol solutions were generally better tolerated than some disinfectant surfactants. It should be mentioned that these latter findings were carried out using sodium laureth sulphate surfactant that was more aggressive to the skin than the non-ionic surfactants used in the present study.

The combined application of liquid soap and alcohol-based gel showed a good skin tolerance. Tolerance was nevertheless lower than that of the liquid soap and alcohol gel used separately. One hypothesis, already raised in the literature [2], suggests that surfactants used initially damage the lipid structure of the SC, making it possible alcohol to infiltrate deeper SC layers. One single combined application of the two offending products, however, presents a better antiseptic potential, and thus is recommended when aseptic conditions are mandatory [3].

The beforehand use of SPC 1 in irritancy tests showed an uncertain benefit. Indeed, a decreased skin tolerance was observed following its use before application of the liquid soap. This decline was accentuated when the alcohol-based gel was further applied and indicated a marked potential irritant effect when using the liquid soap and the alcohol-based gel combination. Interestingly enough, the three products (SPC 1, soap and gel) used singly were better tolerated than when applied in combination. In fact, some interactions between products can generate compounds presenting irritation properties [7,13]. Therefore, it was already reported that a given BC should be specific to the specific irritants.

Positive aspects of SPC use are acknowledged, but some negative effects can occur [7-9]. Their effects depend on their composition and galenic presentation. For instance, preservatives and fragrances [8] can induce irritation and allergic contact dermatitis. In our study, SPC 1 contained parabens as preservatives (Table 1). They are responsible for some allergic potential [4]. The choice of the emulsifiers can also influence the irritant character of the SPC. In SPC 1, propylene glycol may be responsible for some irritation properties [31]. It is also reported that hydrophobic or hydrophilic properties of SPC could increase the skin penetration of irritant substances^7 ^and thus accentuate their harmful effects.

Contrasting with SPC 1, SPC 2 showed a benefit when applied beforehand the application of the liquid soap and the alcohol-based gel. SPC 2 presented a barrier effect against potentially irritant products. Its protective effect can be allotted to its composition in various ingredients including Butyrospermum parkii exhibiting a strong hydrating capacity [32], and glycerol known to be a effective humectants [33], and emollients.

It is likely that the combination of the 2 offending products used in the present study alter both the SC lipids and the skin barrier function [34]. The SPC are expected to exert a preventive effect that is different from products aiming at the skin barrier repair after degradation [35-37].

## Conclusion

This study highlights that corneoxenometry and corneosurfametry bioessays may be conveniently used to compare the protection afforded by SPC application against irritant compounds to the skin. They avoid animal testing and toxicological hazards in human testing. They are cheap, rapid and reproductible. In this study, they were used in conditions relevant with the conditions experienced in medical settings.

Contrasting effects were obtained with the two BC suggesting that a protective effect may be quite specific for the chosen noxious products. Any BC can be protective against a given type of irritant, and conversely inactive against other types of irritants or even increasing the irritant properties of products depending on generated biochemical interactions.

## Competing interests

The authors declare that they have no competing interests.

## Authors' contributions

EXU carried out bioinstrumental assessments and drafted the manuscript, EM collected data, RD participated in the data management, CC participated in the design of the study, GEP conceived the study and participated in its design and coordination. All authors read and approved the final manuscript.
